# Temperature and Humidity PID Controller for a Bioprinter Atmospheric Enclosure System

**DOI:** 10.3390/mi11110999

**Published:** 2020-11-12

**Authors:** Manuel Matamoros, J. Carlos Gómez-Blanco, Álvaro J. Sánchez, Enrique Mancha, Alfonso C. Marcos, J. Pablo Carrasco-Amador, J. Blas Pagador

**Affiliations:** 1 Department of Graphic Expression, School of Industrial Engineering, University of Extremadura, 06006 Badajoz, Spain; ajs@unex.es (Á.J.S.); acmarcos@unex.es (A.C.M.); jpcarrasco@unex.es (J.P.C.-A.); 2 Jesús Usón Minimally Invasive Surgery Centre, 10002 Cáceres, Spain; emancha@ccmijesususon.com (E.M.); jbpagador@ccmijesususon.com (J.B.P.)

**Keywords:** bioprinting, Proportional Integral Derivative control (PID), atmospheric enclosure, temperature, humidity

## Abstract

Bioprinting is a complex process, highly dependent on bioink properties (materials and cells) and environmental conditions (mainly temperature, humidity and CO_2_ concentration) during the bioprinting process. To guarantee proper cellular viability and an accurate geometry, it is mandatory to control all these factors. Despite internal factors, such as printing pressures, temperatures or speeds, being well-controlled in actual bioprinters, there is a lack in the controlling of external parameters, such as room temperature or humidity. In this sense, the objective of this work is to control the temperature and humidity of a new, atmospheric enclosure system for bioprinting. The control has been carried out with a decoupled proportional integral derivative (PID) controller that was designed, simulated and experimentally tested in order to ensure the proper operation of all its components. Finally, the PID controller can stabilize the atmospheric enclosure system temperature in 311 s and the humidity in 65 s, with an average error of 1.89% and 1.30%, respectively. In this sense, the proposed atmospheric enclosure system can reach and maintain the proper temperature and humidity values during post-printing and provide a pre-incubation environment that promotes stability, integrity and cell viability of the 3D bioprinted structures.

## 1. Introduction

Bioprinting is a booming technology that could revolutionize regenerative medicine [1]. It has a specific application in medicine of traditional additive manufacturing technology that superposes layers of material to build a biological structure. Although there are several bioprinting techniques, all of them present some common challenges to be solved, such as cell death during and after bioprinting, a long bioprinting time or insufficient micro-vascularization, among others [2]. Bioinks are commonly cell-laden hydrogels due to their good biocompatibility, so they present highly hydrated 3D networks, such as extracellular matrix, that promote oxygen and nutrient interchange [3].

It is important to know that hydrogels are very sensitive to external changes (e.g., temperature or humidity) because of their high-water content (80–90% *w*/*v*). For this reason, several processes can be associated to uncontrolled bioprinting conditions. Some of them are related to the stability and integrity of 3D bioprinting structures [4] and other ones are focused on the survival of cells during and after the bioprinting process [5]. In the first block, drying hydrogels will increase the concentration of macromolecules and will promote their crowding [3]. Furthermore, the rheological properties of hydrogels are affected by temperature, among other parameters [6]. These two problems are usually controlled during the printing process inside of the bioink cartridge, but unfortunately, the post-printing stage can suffer similar atmospheric-related problems that are currently uncontrolled inside of the current bioprinter enclosure. Hence, the proposed atmospheric enclosure system for bioprinters expects to fill this gap, increasing post-printing stability and, consequently, allowing a higher resolution of the 3D bioprinted structures [7]. In the second block, cell viability can be affected during both the printing and post-printing processes. In the same way, good cell conditions during the bioprinting process are controlled by bioink cartridges, which are out of the scope of this work [8]. However, the control of the atmospheric enclosure inside of bioprinters to assure proper conditions in the post-printing stage, promoting high cell viability, is the other main interest of this work [9].

As far as the authors know, no previous studies have focused on designing, developing and testing an atmospheric enclosure system for bioprinting that could control these critical parameters to assure the integrity and stability of the 3D structure together with the viability of its cells during the post-printing stage. Instead of generating an atmospheric enclosure system, other authors propose to control these atmospheric parameters by reducing bioprinting time, bioprinting into a water-based bath or reducing air flow [3]. Commercial bioprinters, such as Cellink BioX^®^ (Cellink; Boston, MA, USA), Poietis^®^ (Poietis; 33600 Pessac, France) or 3D-Discovery BioFactory^®^ (REGENHU; 1690 Villaz-Saint-Pierre, Switzerland), control the air flow to minimize biological contamination using High Efficiency Particle Arresting (HEPA) filters [10,11,12]. However, none of them currently solve these issues and the 3D bioprinting process is still relatively slow [7], so large 3D structures can greatly benefit from our proposed post-printing control of atmospheric conditions, which could turn bioprinters into a temporal bioincubator while the bioprinting process is ongoing [7,13].

Proportional integral derivative (PID) controllers are commonly used in atmospheric enclosures of other fields, e.g., neonatal [14,15,16] or egg/bird [17,18] incubators. Each different application requires complex or simple adaptations of their mathematical models, according to the factors involved in each of these environments. On the one hand, large enclosures, such as greenhouses [19,20], animal buildings [21] or large spaces [22], must consider many factors: air convection, wall heat loses, inner heat, humidity generation or solar radiation. On the other hand, small enclosures, such as neonatal [14,16,23] or egg/bird [15,24] incubators, can considerer negligible some of these previous factors, but they present a high cross-coupling effect among their variables [25]. For this reason, some studies have focused their interest only on temperature [15,16,23] or just merge two variables, such as temperature and humidity [14,24,26]. Temperature and humidity are highly related variables, so their simultaneous control is complex and requires a fine tuning of the PID parameters [27]. Additionally, both temperature and humidity are significantly influenced by CO_2,_ which increases the complexity of PID controllers due to the cross-coupling effects among these three variables [28,29]. It has been observed that the structural stability of Type I collagen cannot be ensured after the bioprinting process without a controlled 37 °C environment [7]. With this in mind, the CO_2_ variable was left out of the scope of this work for two main reasons. Firstly, it is not expected that cells produce significant CO_2_ emissions during post-printing, so its concentration should remain stable in the global system. Additionally, the cross-coupling interaction of CO_2_ with temperature and humidity will make difficult the optimization of the PID parameters and, consequently, will decrease the robustness and stability of the final PID controller. Hence, we focus our interest on developing a stable and robust PID controller of the two main variables involved in the post-printing stage of bioprinting—temperature and humidity.

Therefore, the main objective of this work is the design, development and validation of a temperature and humidity PID controller for an atmospheric enclosure system with bioprinting purpose. This atmospheric enclosure system could control the post-printing stage independently of the bioprinting technique used. For this purpose, theoretical, simulated and experimental studies have been performed to assure a proper functioning of the proposed system.

## 2. Materials and Methods

### 2.1. Atmospheric Enclosure System Design

The designed atmospheric enclosure system is a sub-divided parallelepipedal with different areas: a bioprinting sub-chamber, a climatic conditions generation sub-chamber and an electronic/mechanic components sub-chamber. All designing was done using Autodesk Inventor^®^, and different area dimensions are shown in Table 1.

**Figure 1:**
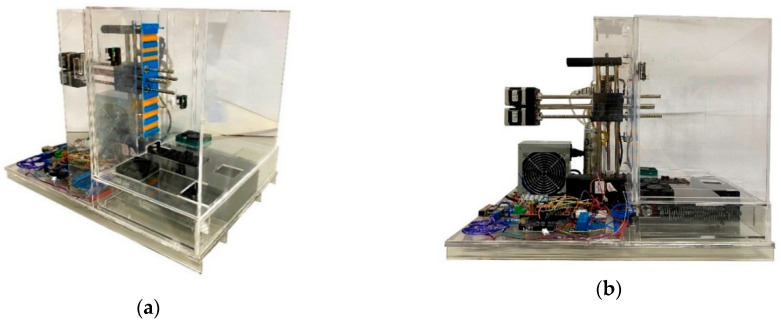
Atmospheric enclosure top (**a**) and lateral (**b**) view.

**Figure 2:**
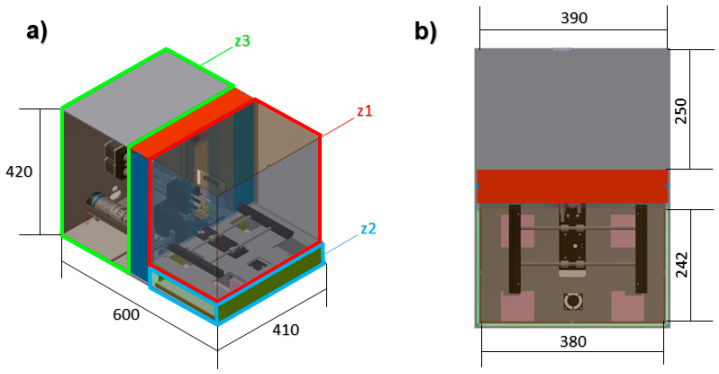
Atmospheric enclosure system tridimensional design: (**a**) perspective and (**b**) top view, where the bioprinting sub-chamber (z1), the climatic conditions generation sub-chamber (z2) and the electronic and mechanical components sub-chamber (z3) are located.

Figure 1 shows the atmospheric enclosure system prototype with its atmospheric enclosure made of methacrylate. Different methacrylate widths were used in each one of the areas to control heat insulation and minimize conduction heat losses. Figure 2 shows the different sub-chambers that make up the atmospheric enclosure system.

Bioprinter control is divided into three different processes: bioprinter mechanical control, atmospheric enclosure control and data visualization. All three processes were individually controlled by Arduino^®^ (Arduino s.r.l.; 20900 Monza, Italy) UNO boards, all connected to a Raspberry^®^ (Rapsberry Pi Foundation; Cambridge CB2 1NF, UK) Pi 3, and the whole system was controlled by a personalized Python^®^ version 3.9 (Python Software Foundation; Wilmington, DE, USA) script. This script provides the user full live control over the bioprinter atmospheric enclosure parameters while temperature, humidity and CO_2_ data are shown.

The control of proper atmospheric conditions inside the enclosure needs generation and sensorization of each one of the inner parameters. In this sense, temperature is generated with two 200-W electrical finned resistances, humidity is produced with cold vaporization of water in a tank using a piezoelectrical transducer and, finally, CO_2_ is injected from an external AquaMedic^®^ (AB Aqua Medic GmbH; Bissendorf, Germany) CO_2_ pressurized bottle when a Blau^®^ (Barcelona Marine Farm S.L.; Barcelona, Spain) (3VA, 14mA, IP 85) electro valve is opened. Likewise, the type and location of every one of the sensors used is shown in Table 2. Generation electronics is switched ON/OFF by a TONGLING (Xiamen Hongfa Electroacoustic Co; 361021 Xiamen, China) JQC-3FF-S-Z relay moduli.

**Figure 3:**
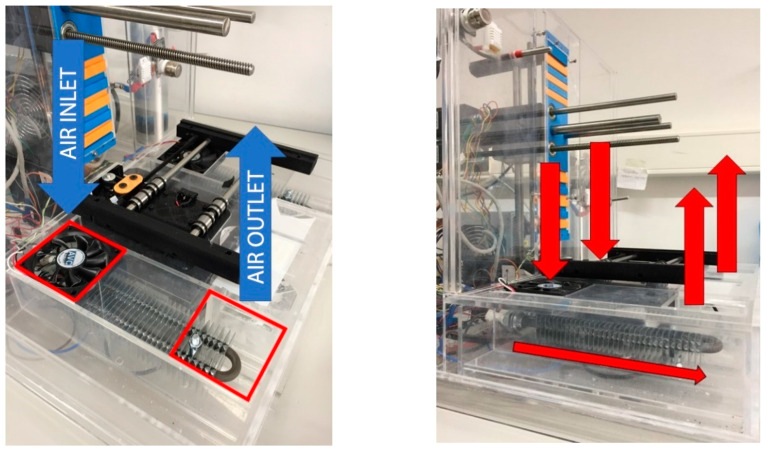
Air inlets/outlets schema.

The main area of the atmospheric enclosure system is the climatic conditions generation sub-chamber. All generation apparatuses were placed in this area to produce the appropriate atmospheric conditions. This sub-chamber is a separated area of the enclosure connected by 4 air inlets/outlets, 2 inlets with axial fans and 2 outlets, as shown in Figure 3. The working principle of this area is to force the air to enter into the generation sub-chamber where its temperature, humidity and CO_2_ are modified and then exit to the enclosure atmosphere again. This process is continuously repeated, creating an air conditioning flow until the atmospheric parameters’ target values are reached.

To ensure a sterile environment at the beginning of the bioprinting process, the bioprinter sub-chamber is sterilized by action of four UV LED panels (420 nm and 440 mW) incorporated in the bioprinter sub-chamber. After this process, air is introduced into the system from outside, passed through a HEPA filter and generates positive pressure to prevent the entry of contaminating external agents.

Although the proposed atmospheric enclosure system has a CO_2_ pressurized bottle and two CO_2_ sensors for future purposes, they have not been used in this study. 

### 2.2. Mathematical Modelling

The purpose of this work is to control the inner temperature and humidity of an atmospheric enclosure system. In this sense, the inner heat balance and water mass balance equations are the general non-lineal differential equations used in temperature and humidity control processes [21,22]. In this work, terms with a negligible interaction in the total balance were not considered. Therefore, our heat and water mass balance equations [20] were given by:

(1) $$C_{v}V\frac{dT_{h}}{dt}=Q_{in}-Q_{out}=\left(G_{a}\rho C_{p}T_{c}+Q_{n}\right)-\left(G_{a}\rho C_{p}T_{h}+\frac{T_{h}-T_{o}}{R}\right)$$

(2) $$\rho V\frac{d\left(d_{n}\right)}{dt}=G_{a}\rho d_{c}+D_{n}-G_{a}\rho d_{n}$$

where *C_v_* is the volumetric air heat capacity (J/kg∙°C); *V* is the inner air volume (m^3^); *T_h_* is the inner temperature (°C); *G_a_* is the air mass flow that a fan extracts from the chamber to the generation sub-chamber (m^3^/s); ρ is the air density (1.25 Kg/m^3^); *C_p_* is the air-specific heat (1006 J/kg∙°C); *T_c_* is the generation sub-chamber air temperature (°C); *Q_n_* is the heat dissipation capacity (J/s); *T_0_* is the external temperature (°C); *R* is the enclosure thermal resistance (°C∙s/J); *d_n_* is the inner humidity (g/kg); *d_c_* is the external humidity(g/kg); *D_n_* is the enclosure humidity gain (g/s).

Transfer functions for the PID controller were obtained applying the Laplace transform to Equations (1) and (2). Defining *X* as $\frac{1}{G_{a}\rho C_{p}+\frac{1}{R}}$ these functions are:

(3) $$G_{1}\left(s\right)=\frac{T_{h}\left(s\right)}{T_{c}\left(s\right)}=\frac{K}{1+Ts}=\frac{G_{a}\rho C_{p}X}{1+\left(C_{v}VX\right)s}$$

(4) $$G_{2}\left(s\right)=\frac{d_{n}\left(s\right)}{d_{c}\left(s\right)}=\frac{1}{1+T_{1}s}=\frac{1}{1+\frac{V}{G_{a}}s}$$

where *G*_1_(*s*) and *G*_2_(*s*) are temperature and humidity terms, respectively. 

### 2.3. Experimental Evaluation

A comparative between theoretical and experimental behaviors (theoretical and experimental transfer functions) of the atmospheric enclosure was proposed. For this purpose, the different experimental behaviors of each parameter (inner temperature and humidity) were tested and determined. To obtain data for the experimental transfer function, single input, single output (SISO) and multiple inputs, multiple outputs (MIMO) tests were performed as follows:

1. SISO test to study inner temperature when heater (electric resistance) was switched on.
2. SISO test to study inner humidity when humidifier (cold mist humidifier) was switched on.
3. MIMO test to study coupling of inner temperature and humidity [24,29].

Initial inputs and target values for all tests are shown in Table 3. Atmospheric values have been chosen based on criteria consulted in the literature [30].

Experimental transfer functions were obtained from test data using the Matlab^®^ R2018b System Identification Toolbox™. This tool is commonly used to obtain mathematical models of dynamic systems from experimental input and output data. As such, Matlab^®^ R2018b Simulink™ was used to simulate and compare both theoretical and experimental transfer functions.

### 2.4. PID Controller

Variations in temperature and humidity can change bioprinting materials’ properties and affect the cellular viability, stability and integrity of 3D bioprinted structures. To minimize variations, a PID controller was added to the atmospheric enclosure system. PID controller algorithms are widely used in feedback control systems and are mathematically expressed as [31]:

(5) $$u\left(t\right)=K\left(e\left(t\right)+\frac{1}{T_{i}}\overset{t}{\underset{0}{\int }}e\left(\tau \right)d\tau +T_{d}\frac{de\left(t\right)}{dt}\right)$$

**Figure 4:**
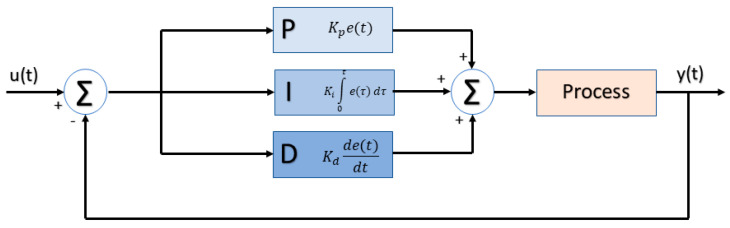
Atmospheric enclosure block diagram with a proportional integral derivative (PID) controller.

A good PID control system can provide the atmospheric enclosure with the necessary stability in the shortest time. In this sense, the Ziegler–Nichols closed-loop method was used in Matlab^®^ R2018b (MathWorks; Natick, Massachusetts, United States) to tune all PID parameters using process input/output signals. All these calculations were done through a simulation block diagram implemented in Matlab^®^ R2018b Simulink™, which follows the standard structure shown in Figure 4.

### 2.5. Procedure

The workflow was performed as Yinping et al. [28] in their work. First, mathematical model behavior equations were solved, obtaining all constants needed to calculate the theoretical system transfer functions. Second, temperature and humidity transfer functions were calculated and evaluated with experimental transfer functions obtained from MIMO and SISO tests. Several behavior approaches of the system were obtained using the least-square method in the Matlab^®^ R2018b System Identification Toolbox™. All possible types in this toolbox were used: one pole (P1), two poles (P2), three poles (P3), one pole and one zero (P1Z), two poles and one zero (P2Z), three poles and one zero (P3Z), two poles sub-damped (P2U), three poles sub-damped (P3U), two poles and one zero sub-damped (P2ZU), three poles and one under-damped zero (P3ZU), one pole and one integrator (P1I), two poles and one integrator (P2I), three poles and one integrator (P3I), one pole and one delay (P1D) and two poles and one delay (P2D) transfer functions.

Next, both transfer functions’ (theoretical and experimental) block diagrams were designed and evaluated. Finally, theoretical and experimental transfer functions were analyzed, obtaining the first Kp (proportional controller), Ki (integral controller) and Kd (derivative controller) values using the Ziegler–Nichols method. Then, this first PID tuning was tested and manually re-tuned to limit overshoots produced by the Ziegler–Nichols method.

To validate the atmospheric enclosure system, several tests were performed using the former calculated Kp, Ki and Kd values. Finally, to check the quality of calculated PID, an experimental error (6) was calculated.

(6) $$\varepsilon_{r}=\frac{\left|V_{real}-V_{model}\right|}{V_{real}}\cdot 100$$

## 3. Results

The following temperature (7) and humidity (8) theoretical transfer functions were obtained once inherent constants of the system were replaced in Equations (3) and (4).

(7) $$G_{1}\left(s\right)=\frac{T_{h}\left(s\right)}{T_{c}\left(s\right)}=\frac{0.839}{1+0.263\;s}$$

(8) $$G_{2}\left(s\right)=\frac{d_{n}\left(s\right)}{d_{c}\left(s\right)}=\frac{1}{1+0.557\;s}$$

### 3.1. SISO Test to Study Inner Temperature When Heater (Electric Resistance) Was Switched on

The experimental transfer function for temperature was obtained from a SISO test with laboratory environmental conditions set to 22 °C and 50% relative humidity. In the tests, actuators (electric heat resistance) were powered on or off when the inner temperature registered by sensors was below or above the target temperature (37 ± 1 °C). Data acquisition from sensors takes 700 s (test time) to assure a complete study with enough data.

**Figure 5:**
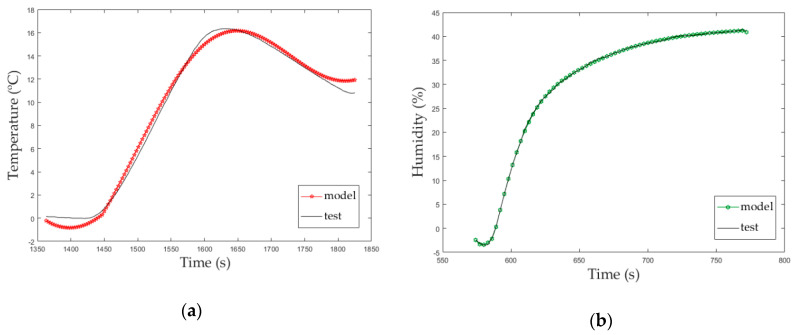
(**a**) Single input, single output (SISO) temperature study; (**b**) SISO humidity study.

The experimental transfer function, Equation (9), is calculated from the selected fitting plot for electric heater switching, the one pole and one delay (P1D) plot (Figure 5a). The accuracy rate calculated with Matlab^®^ R2018b for this transfer function was 99.49%.

(9) $$G_{t,exp}\left(s\right)=\frac{Kp}{1+\mathrm{Tp}1\cdot s}=\frac{2.55\cdot e^{-42\;s}}{1+576.74\;s}$$

### 3.2. SISO Test to Study Inner Humidity When Humidifier (Cold Mist Humidifier) Was Switched on

Using previously described methodology for SISO tests, the plot that best fit the humidity behavior (Figure 5b) was one pole and one delay (P1D). Equation (10) is the experimental transfer function for humidity calculated from P1D plot. The accuracy rate calculated with Matlab^®^ R2018b for this transfer function was 99.03%.

(10) $$G_{h,exp}\left(s\right)=\frac{Kp\cdot e^{-Td\cdot s}}{1+\mathrm{Tp}1\cdot s}=\frac{1.05\cdot e^{-10.26\;s}}{1+48.83\;s}$$

A slightly variation between the experimental and theoretical transfer functions (Equations (7) and (9) vs. Equations (8) and (10)) can be observed; an exponential function is multiplied to the proportional constant. Albright et al. [25] explain in their work that temperature and humidity sensors usually need this exponential function to control what they called dead time (Td), meaning a significative delay in the sensor measurement.

Figure 5 shows a comparison between the experimental data (test) and the Matlab^®^ R2018b model from these data that extract the transfer function with a specific accuracy (model). These graphs represent the first approach of the solution that will be used as input of Section 3.3 and, after that, as initial values of the MIMO test to build a decoupled feedback of the atmospheric enclosure system.

### 3.3. Comparative between Theoretical and Experimental Transfer Functions

**Figure 6:**
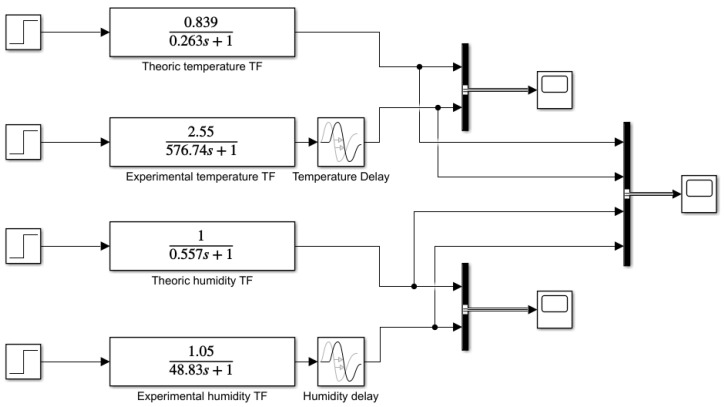
Comparative transfer functions implemented using a Simulink block diagram.

**Figure 7:**
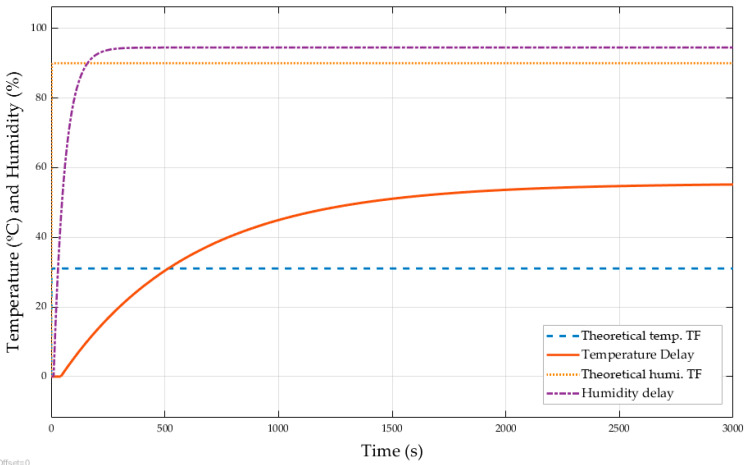
Theoretical and experimental transfer functions simulation without a PID controller.

An open-loop block diagram (Figure 6) to compare theoretical and experimental SISO transfer functions before adding the PID controller was designed in Matlab^®^ R2018b Simulink™. Additionally, two delay blocks have been added to this diagram to simulate the delay of sensors while reading in experimental functions. Table 3 shows all values used in inlet steps. As can be seen in Figure 7, without the addition of a controller, both temperature and humidity surpass the target values and stabilize at a higher value, taking 2500 and 250 s, respectively.

### 3.4. MIMO Test to Study Coupling of Inner Temperature and Humidity

**Figure 8:**
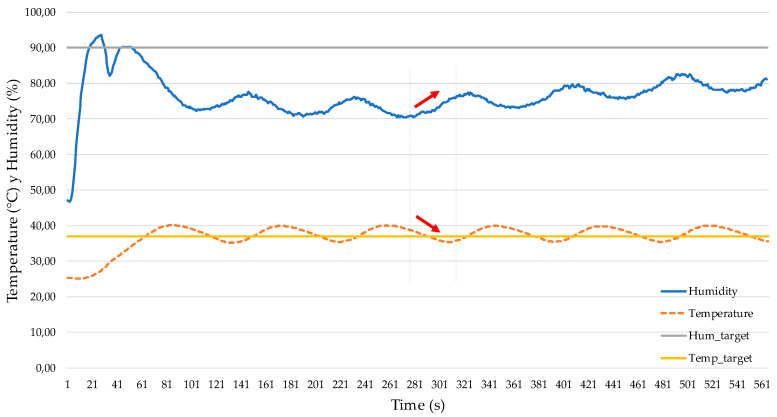
Humidity behavior against temperature without a PID controller.

The dependency between temperature and humidity means that control could be a hard task when they are actuating at the same time. Wang et al. [24] exposed a higher influence of temperature on relative humidity and vice versa. They also observed a decrease by about 2–3% of relative humidity when temperature increases by 1 °C. Figure 8 shows this influence of temperature on humidity in our atmospheric enclosure system before the PID controller was set. It can be seen, without the PID controller, that both temperature and humidity fluctuate in a wide range.

Analyzing Figure 8 plots, the system responses to some characteristic parameters of the atmospheric enclosure system are exposed in Table 4.

**Figure 9:**
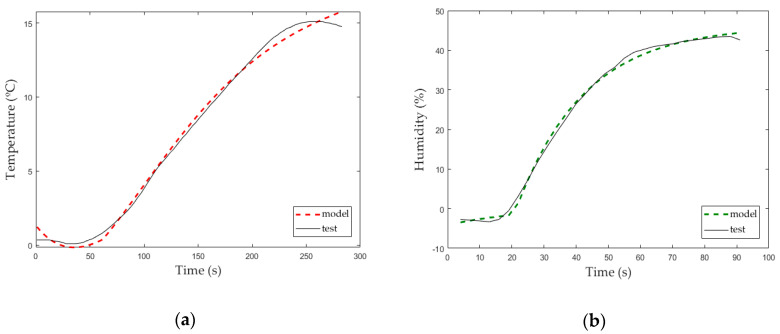
(**a**) SISO temperature study, (**b**) SISO humidity study.

Using the before-mentioned methodology, a MIMO test was performed on the atmospheric enclosure system to obtain the inherent values of the system. Laboratory environmental conditions were set to 22 °C and 50% relative humidity and the target values were the same as those used in the previous MIMO tests. Analyzing all obtained plots, P1D with delay models were the ones that best fit the temperature and humidity behaviors (Figure 9). The associated transfer functions are described in Equations (11) and (12), with a 95.01% and 99.42% accuracy rate, respectively.

(11) $$G_{t+h,exp}\left(s\right)=\frac{Kp}{1+\mathrm{Tp}1\cdot s}=\frac{2.55\cdot e^{-57.89\;s}}{1+576.74\;s}$$

(12) $$G_{h+t,exp}\left(s\right)=\frac{Kp\cdot e^{-Td\cdot s}}{1+\mathrm{Tp}1\cdot s}=\frac{1.05\cdot e^{-13.62\;s}}{1+48.83\;s}$$

Figure 9 shows a comparison between the experimental data (test) and the Matlab^®^ R2018b model from these data that extract the transfer function with a specific accuracy (model). These graphs represent a former approach to the final solutions that will integrate other elements, such as the response time of the sensor, which will be exposed in Section 3.4.

### 3.5. Obtaining PID Values and Modeling System in Matlab/Simulink™ 

Previous results of our theoretical transfer functions cannot be considered good enough to describe our system. Interrelations between variables, non-linearity, high hysteresis or real-time variations are some reasons why temperature and humidity are complex systems to control. In this sense, performing temperature and humidity tests without any kind of control system variables will provoke high fluctuation and poor stabilization of the system. Therefore, to design a good PID controller, natural fluctuations of variables were considered in the new system modelling.

Using the Matlab^®^ R2018b Pidtool toolbox, PID controller values of transfer functions (11) and (12) were obtained. This tool provides two different PID behavior adjustments: response velocity and long-time stabilization. PID values (Table 5) were obtained by setting a fast and robust response to the controlled system.

**Figure 10:**
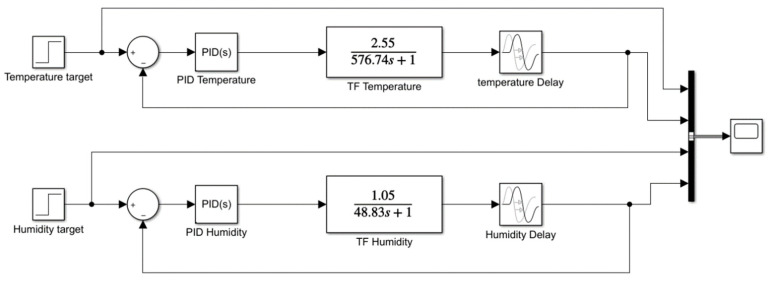
System block diagram with temperature and humidity.

The block diagram shown in Figure 10 uses the fitted PID values in Table 5 and compares the modelled and the real system behaviors in Matlab^®^/Simulink™.

**Figure 11:**
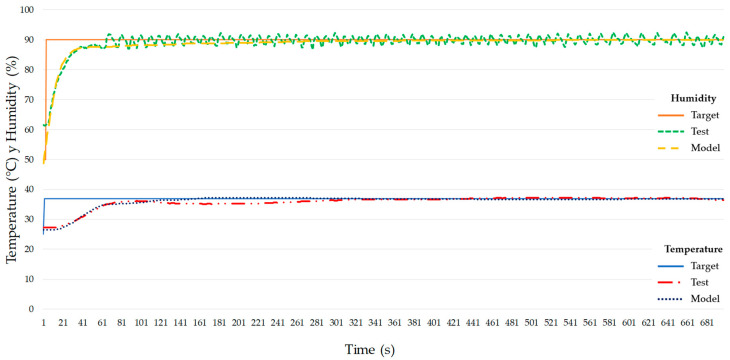
Theoretical and experimental simulated system using a decoupled approach for the PID controller.

Results from the experimental test using a decoupled PID controller of temperature and humidity can be seen in Figure 11.

**Figure 12:**
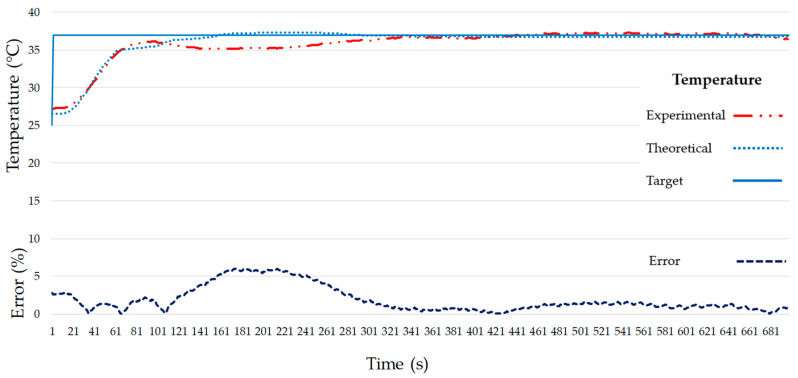
Target, model and experimental temperature (**top**) and experimental temperature error (**bottom**) of the final atmospheric enclosure system.

**Figure 13:**
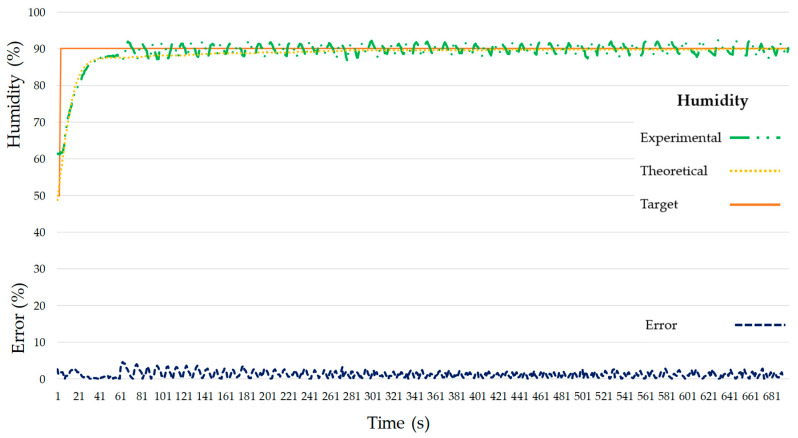
Target, model and experimental humidity with experimental humidity error.

The temperature behaviors for the theoretical and the real system are very close. Figure 12 shows a maximum error for temperature of 6.01% at 185 s and the system has an average error of 1.89% once stabilized. Regarding humidity, there is a maximum error of 4.59% at 68 s, then the humidity value oscillates around the target value with an average error of 1.30% (Figure 13). The stabilization times for the theoretical models were 161 s for temperature and 281 s for humidity. Despite temperature stabilizing faster than humidity for the theoretical models, the stabilization times achieved with the proposed PID values (test values) were 311 s for temperature and 65 s for humidity. Maybe this is due to a higher error (6.01%) of the temperature model as opposed to a 1.89% error of the humidity model. Matlab^®^ R2018b calculates the theoretical transfer function for the temperature with a 95.01% accuracy that can justify these differences between the theoretical and experimental values.

## 4. Discussion

The objective of this work is to determine the optimum PID controller values of an atmospheric enclosure system for bioprinting. Specifically, we propose to control temperature and humidity to improve the integrity, stability and cell viability of 3D bioprinted structures. In order to design and develop our atmospheric enclosure, three components of the system were analyzed and compared with previous studies: the mathematical model, the transfer functions and the block diagram, but it was also analyzed whether to use coupled or uncoupled variables. 

The similarity between the proposed atmospheric enclosure system and greenhouses or animal buildings suggests that the mathematical model used by Daskalov et al. [20] and Albright et al. [25] (heat and humidity balances) can be used in our system after some important adaptations. In this sense, the presence of animals in the study of Daskalov et al. [20] requires some factors in the mathematical model, such as sensitivity to heat or water steam produced by animals and the heat lost due to mechanical ventilation. All of these factors must be eliminated for our bioprinting environment. In addition, Albright et al. included terms of solar radiation heat, ventilation volumetric flow and plant evapotranspiration, which have no purpose in our mathematical model. Lastly, our transfer function calculations from differential equations were based on Yinping et al.’s [28] work, using a similar structure for transfer functions relating to the behavior of temperature and humidity.

According to the bibliography, there is no agreement about the best way to control temperature and humidity. So, some authors propose coupled controllers [28,32,33] while others propose decoupled controllers [24,25,34]. In this sense, knowing that an increase of 1 °C can decrease humidity by about 2–4 % [24], we performed SISO and MIMO tests to study each parameter independently and simultaneously, respectively. Considering our design and the MIMO test results (Figure 8), we finally decided to design a decoupled temperature and humidity controller that produces better results with simple PID calculations as well as increasing stability and robustness of the goal system [24]. 

Once the type of controller was chosen, the next step was the composition of block diagram, whose main element will be the system’s plant transfer function for temperature and humidity. Previous studies proposed different types of transfer function to control temperature and humidity: some of them without delay [14,32] and others considering delay [24,34,35], which is a parameter totally dependent on the technical specifications (response time) of the electronic components [21]. Those authors that proposed no delay transfer functions try to simplify the system interactions, but their models are supposed to be less real [17,25,34]. On the other hand, the delay is commonly considered when complex transfer functions with high oscillatory responses and difficulties in stabilizing disturbances are present [25]. Hence, our system provides a better response using delay, specifically P1D type, similar to other previous studies [24,34,35].

Regarding the block diagram, three different blocks have been used: control, plant and delay. Although this diagram is quite common [25], in the same way as transfer function, delay inclusion or exclusion will modify the performance of the PID controller. So, some studies excluded the delay for temperature [17,22,28] while others considered it in their schemes for temperature and humidity [32,36]. In our case, an adapted version of Yiping et al. [28] that considers delays has been used, but other complex approaches with predictive algorithms should be considered for future improvements [36].

Finally, the global performance of our atmospheric enclosure system is pretty good. In this sense, low stabilization time and errors were obtained in the experimental tests. Other authors presented a temperature stabilization time of 2760 s for a 4 °C increment [14], 600 s for a 10 °C increment [34] or 246 s for a 5 °C increment [33]. Nevertheless, our enclosure system can stabilize a 12 °C temperature increment in 311 s. Regarding humidity, previous stabilization times were 420 s for a 10% increment [33] or 300 s for a 5% increment [34]. However, our system can stabilize a 40% humidity increment in only 65 s. It is important to note that different settings, such as heat devices, humidifiers or enclosure volume, can unfairly bias this comparison. For this reason, a specific comparative study should be performed when other atmospheric enclosure systems will be available for bioprinting. Meanwhile, we have exposed some differences among our work and the previously exposed studies for better understanding of our previous comparisons. In some cases, no technical specifications of sensors/devices are available; in this case, we cannot go into detail about the reasons of differences [34]. Other studies with a larger volume of controllers and heaters/sensors are differentially dimensioned [33]. Maybe the most similar enclosure was an incubator with comparable electronics, but unfortunately, the authors did not control humidity in the analysis [15].

## 5. Conclusions

In this work, a PID controller for temperature and humidity control of an atmospheric enclosure system for bioprinting was designed, developed and tested. Theoretical and experimental transfer functions for temperature and humidity were calculated and verified. The results show that the proposed atmospheric enclosure system is capable of stabilizing temperature and humidity in 311 s and maintaining target values with an average error of 1.89% and 1.29% for temperature and humidity, respectively. Hence, the proposed atmospheric enclosure system for bioprinting could improve post-printing environmental conditions to increase the integrity, stability and cell viability of 3D bioprinted structures. 

As commented, to guarantee a proper atmospheric enclosure system for bioprinting, a CO_2_ control is needed in addition to temperature and humidity. In this sense, the next step of this study will be analyzing the system’s behavior after the inclusion of carbon dioxide as well as the interrelation with temperature and humidity. Additionally, bioprinters usually have a heat/cool cartridge in the extruder to control the bioink temperature. So, the addition of this heat/cool source inside the atmospheric enclosure to simulate bioprinter performance while extruding will be another future development.

## Figures and Tables

**Table 1:** Atmospheric enclosure dimensions.

Areas of the Atmospheric Enclosure	Size (mm)
Atmospheric enclosure full size	600 × 410 × 420
Bioprinting sub-chamber (z1)	380 × 242 × 340
Climatic conditions generation sub-chamber (z2)	380 × 242 × 75
Electronic and mechanical components sub-chamber (z3)	390 × 250 × 410

**Table 2:** Atmospheric enclosure sensors.

Sensors	Designation	Position
2× Temperature sensor	PT 100 PRO	Top left and bottom right
2× Humidity sensor	DHT22	Top left and bottom right
2× CO_2_ sensor	MG811	Top left and bottom right

**Table 3:** Initial inputs and target values used in tests.

Variable	Input Value	Target Value
Temperature	25 °C	37 °C
Humidity	50%	90%

**Table 4:** Atmospheric enclosure system responses to some characteristic parameters.

Value (s)	Temperature	Humidity
Delay time	16.00 s	6.00 s
Rise time	40.00 s	11.00 s
Peak time	85.00 s	26.00 s
Percent overshoot	8.35%	3.88%
Settling time	155.00 s	106.00 s

**Table 5:** PID values obtained.

Value	Temperature (°C)	Humidity (%)
Proportional (Kp)	150.00	25.00
Integral (Ki)	1.00	2.00
Derivative (Kd)	6000.00	100.00
